# The Role of Online Support Groups in Helping Individuals Affected by HIV and AIDS: Scoping Review of the Literature

**DOI:** 10.2196/27648

**Published:** 2022-07-26

**Authors:** Neil S Coulson, Heather Buchanan

**Affiliations:** 1 School of Medicine Faculty of Medicine and Health Sciences University of Nottingham Nottingham United Kingdom

**Keywords:** AIDS, HIV, online support groups, internet, peer support, social support, synthesis, systematic review

## Abstract

**Background:**

Online support groups provide opportunities for individuals affected by HIV and AIDS to seek information, advice, and support from peers. However, whether and how engagement with online support groups helps individuals affected by HIV and AIDS remains unclear, as does the nature of the evidence on this topic.

**Objective:**

This scoping review sought to explore whether engagement with HIV and AIDS–related online support groups benefits members in terms of psychosocial well-being and illness management, whether members experienced any negative aspects of these groups, and what types of social support are exchanged within HIV and AIDS–related online support groups.

**Methods:**

A scoping review of English-language articles (including both qualitative and quantitative studies) was undertaken using the PRISMA (Preferred Reporting Items for Systematic Reviews and Meta-Analyses) guidelines. The databases searched included MEDLINE, PubMed, EMBASE, CINAHL, PsycINFO, CENTRAL (Cochrane Register of Controlled Trials), and Scopus. Key findings were synthesized using a narrative and thematic approach.

**Results:**

A total of 22 papers met the inclusion criteria from an initial pool of 3332 abstracts. These papers included 23% (5/22) quantitative studies, 9% (2/22) mixed methods studies, and 68% (15/22) qualitative studies published between 2007 and 2019. Cross-sectional evidence suggests that engagement with HIV and AIDS–related online support groups is empowering for members and may lead to a range of psychosocial benefits. Furthermore, qualitative evidence suggests that these groups provide an opportunity to connect with similar people and share experiences. This can help improve self-worth, reduce stigma, facilitate improved illness management, and gain greater confidence when interacting with health professionals. However, online support groups are not without their limitations as qualitative evidence suggests that users may encounter examples of interpersonal conflict between members as well as be exposed to challenging content. Finally, HIV and AIDS–related online support groups are avenues through which individuals can solicit support, most commonly informational or emotional.

**Conclusions:**

HIV and AIDS–related online support groups may have some benefits for members, particularly in terms of providing social support. There is a need for a systematic review of this literature that includes an assessment of the methodological quality of the available evidence.

## Introduction

### Background

According to the Joint United Nations Programme on HIV and AIDS, it is estimated that approximately 38 million people are living with HIV and AIDS worldwide [1]. Although there is currently no cure, it is possible to suppress the virus to levels that are undetectable using antiretroviral drugs. Nevertheless, a new diagnosis of HIV brings with it several challenges [2,3]. Individuals receiving a positive diagnosis of HIV will likely face a lifetime of medical treatment to combat the biomedical repercussions of the disease. Individuals also face many psychological challenges because of living with a long-term, highly stigmatized condition [4,5], which may lead to uncertainty about the future. Individuals living with HIV and AIDS may experience social isolation [6], fear of prejudice [7], and loneliness [8] as they learn to adjust to their diagnosis and a lifetime of daily multiple medications [9]. If individuals have problems adjusting to living with HIV and AIDS, clinical depression, anxiety, stress, and poor coping are common [10]. Of particular concern is the prevalence of depression and suicide among people living with HIV and AIDS [11-13].

Evidence suggests that individuals living with HIV and AIDS who are satisfied with the level of social support they receive are more likely to adjust positively, cope better, and experience a slower progression of HIV-related symptoms [14]. Furthermore, research has shown that social support plays a pivotal role in managing the stress associated with HIV and leads to better psychological and physical health outcomes among individuals living with the disease [15,16].

As global access to the internet continues to increase [17], recent technological advances have led to the development of diverse forms of electronic communication, which in turn have supported participation, collaboration, and information sharing between users. An example that illustrates the potential for users to interact with peers on the web is through the medium of online support groups. Such groups allow individuals to come together to share experiences, provide mutual support, and ask questions. Online support groups can be underpinned by different platforms such as discussion forums, chat rooms, social networking sites (eg, Facebook), blogs, microblogs (eg, Twitter), and virtual reality environments. Although such platforms may offer synchronous interaction (ie, live and in real time), most online support groups are asynchronous, where interaction and the exchange of user-generated content takes place over time (ie, hours, days, weeks, or months), and are predominantly text based.

In recent years, there has been an exponential increase in the number of online support groups that have been established to help those affected by long-term conditions, including HIV and AIDS. Similarly, the number of internet users accessing online support groups continues to increase, with recent estimates suggesting that between 7% and 28% of adults have accessed one [18,19].

Evidence suggests that there may be a range of factors that can lead individuals to engage with asynchronous text-based online support groups. In a review of the literature, Wright [20] described 4 broad factors evident within the studies. First, the convenience of computer-mediated communication may be attractive to individuals. For example, an asynchronous text-based online support group is potentially available 24 hours per day, 7 days per week and can be accessed whenever it is needed [21]. This flexibility in access permits individuals to seek support at times and places that are convenient to them and may be helpful to those with family, educational, or work commitments [22-24]. Second, individuals may have limited access to adequate social support within traditional social networks. This may be because people in an individual’s social network have little or no experience or understanding of their condition. Indeed, their condition may not be well understood by health professionals, or it may be rare. Third, individuals may be living with a condition that is stigmatized and, therefore, online support groups may be regarded as a safe environment in which they can discuss personal or sensitive issues [25,26]. Fourth, individuals have reported the value of being able to interact with others who are similar and credible [27]. Taken together, it is evident that online support groups may be relevant and potentially beneficial for individuals living with HIV and AIDS.

However, it should be noted that online support groups are not without their limitations. For example, the asynchronous text-based nature of most online support groups means that social cues such as facial expressions, tone of voice, and body language are not available, and this may cause challenges for users [28]. In addition, the absence of physical proximity restricts the expression of physical affection (eg, hugs), and users may feel isolated and alone in their real lives after logging off [24]. There may also be delays in responses being posted to the group, and this may negatively affect the user experience and satisfaction with online support groups [21]. Concerns have also been expressed regarding both the quantity and quality of posts and information [29,30]. Finally, there may exist a dominance of negative content as users are more likely to post messages during times when their symptoms are especially problematic, and this may cause additional anxiety and concern for those reading these posts [24].

### Rationale for the Study

The internet affords new opportunities to support those living with a stigmatized condition such as HIV and AIDS. Online support groups provide a convenient, anonymous, and increasingly popular way to reach out to similar people for information, advice, and support. In addition, increasing attention has been given to online support groups by policy makers [31]. However, there has been no attempt to review the evidence on the possible benefits (or limitations) of HIV and AIDS–related online support groups as well as the types of social support that may be exchanged between users. In this scoping review, both the quantitative and qualitative literature will be identified, described, and synthesized.

### Aim and Review Questions

The primary aim of this scoping review was to describe the literature on the utility of online peer support groups for individuals affected by HIV and AIDS, and 3 key questions informed our review. We did not make any assumptions about whether quantitative or qualitative studies would ultimately be used to address each question. Rather, our scoping review set out to establish what types of evidence existed that could help address each of the research questions. Our questions were as follows: (1) Does engagement with online peer support groups improve psychosocial well-being and illness management among those living with HIV and AIDS? (2) Are there any negative aspects of online support groups experienced by individuals living with or affected by HIV and AIDS? If so, what are these? (3) What types of social support are exchanged within online support groups for individuals living with or affected by HIV and AIDS?

## Methods

### Search Strategy and Procedure

Following the PRISMA (Preferred Reporting Items for Systematic Reviews and Meta-Analyses) checklist, our search strategy protocol was published in PROSPERO (registration CRD42020161119). MEDLINE, PubMed, EMBASE, CINAHL, PsycINFO, CENTRAL (Cochrane Register of Controlled Trials), Scopus, and Google Scholar were searched electronically. The search strategy focused on 2 central concepts: the intervention (ie, online support groups) and the population (ie, individuals living with or affected by HIV and AIDS). It was developed using a combination of Medical Subject Headings and keywords with no study design filter. In addition, the references of the selected articles were hand searched for any additional relevant studies. The searches were conducted in April 2022 (for an example of the MEDLINE search and PRISMA checklist, see Multimedia Appendices 1 and 2).

### Inclusion and Exclusion Criteria

To be eligible for inclusion in our scoping review, an article needed to (1) be peer-reviewed and (2) meet the inclusion criteria detailed in Textbox 1. These criteria were developed using the Population, Intervention, Comparator, Outcome, Setting, and Study Design model. We did not apply any restrictions with regard to date of publication. Our inclusion and exclusion criteria were developed such that we could focus solely on the unique contribution of text-based HIV and AIDS–related online support groups; therefore, we chose to exclude studies in which this support was combined with other forms of support.

**Population, Intervention, Comparator, Outcome, Setting, and Study Design inclusion and exclusion criteria.**

**Population**

- Inclusion: individuals affected by HIV and AIDS either directly (ie, patient) or indirectly (eg, family member, loved one, colleague, or friend)
- Exclusion: individuals not affected by HIV and AIDS either directly or indirectly (eg, health professionals)

**Intervention**

- Inclusion: all types of support groups offered via the internet using either an asynchronous (eg, email listserv, message board, or open or closed social media groups) or synchronous (eg, chat room) text-based platform
- Exclusion: studies that evaluated a *combination* of face-to-face or telephone support with either an asynchronous or synchronous text-based platform

**Comparator**

- Inclusion: studies with or without a comparison or control group
- Exclusion: none

**Outcome**

- Inclusion: studies that reported on engagement with and utility of text-based HIV and AIDS–related online support groups in terms of psychosocial well-being and illness management, studies that reported the types of social support exchanged within text-based HIV and AIDS–related online support groups, and studies that reported negative experiences of engagement with text-based HIV and AIDS–related online support groups
- Exclusion: descriptive studies (eg, sociodemographic profile of users)

**Setting**

- Inclusion: text-based support platforms
- Exclusion: face-to-face setting

**Study design**

- Inclusion: all quantitative designs, qualitative studies that explored participants’ experiences of online support groups as reported directly from the users, and studies reporting the analysis of user-generated content
- Exclusion: literature reviews and microanalysis of online discourse (eg, discourse or conversation analysis)

### Study Selection

The 2 authors (NSC and HB) reviewed the titles and abstracts independently to identify potentially relevant articles. Abstracts not meeting the inclusion criteria were excluded. In cases where the abstract signaled potential eligibility, the full article was retrieved. Inclusion was based on agreement between both authors, and all reasons for exclusion were noted. In all instances of disagreement, discussion took place until the conflict was resolved.

### Data Extraction and Data Synthesis

Predetermined study characteristics (ie, study aim, methods, data source, sample, and data analysis) as well as results (ie, quantitative findings, identified positive or negative outcomes, experiences or attributes, and types and frequencies of social support) were extracted by each of the 2 authors independently. To support this process, the review tool Covidence was used, and the extraction template was modified to support the specific requirements of the scoping review. Each of the 2 authors independently extracted the study characteristics and findings before discussing and agreeing on the final extraction content, which was then entered into a specific section of the Covidence data extraction template. To address each of the research questions, we used both narrative (ie, tabulation of findings from individual primary studies) and thematic (ie, an inductive approach to generate descriptive or analytical themes) synthesis.

## Results

### Included Studies

**Figure 1:**
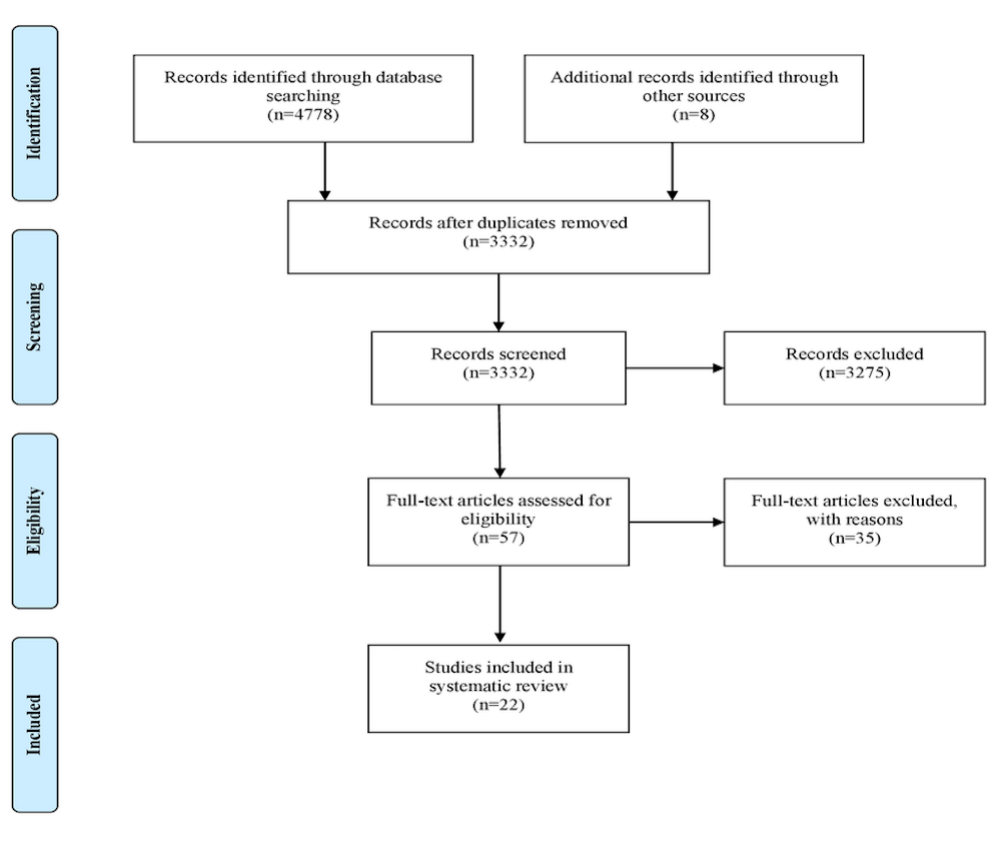
PRISMA (Preferred Reporting Items for Systematic Reviews and Meta-Analyses) flow diagram of the studies included in the review.

Our search strategy yielded 4786 studies, including 1454 (30.38%) duplicates. From the 3332 titles and abstracts reviewed, 2993 (89.83%) did not meet the inclusion criteria. The full texts of the remaining 57 studies were assessed, and a further 35 (61%) were excluded as they did not meet the inclusion criteria (Table 1) or were duplicates (n=11, 19%). Overall, there were 22 papers included in our review. Figure 1 presents the PRISMA flow diagram.

**Table 1:** Reasons for exclusion at the full-text review stage (N=35).

Reasons for exclusion	Studies, n (%)
No outcomes, experiences, or attributes of online support groups reported	14 (40)
Duplicates	11 (31)
Conference abstracts but that contained insufficient data for extraction	2 (6)
Microanalyses of online discourse	2 (6)
Peer support embedded within a complex intervention	2 (6)
Literature review or opinion article or discussion only	1 (3)
Not focused on HIV and AIDS	1 (3)
Focused on health professionals	1 (3)
Not obtainable	1 (3)

### Study Characteristics

For those studies included in the review, further details are provided in Table 2. As indicated, of the 22 studies, 5 (23%) were quantitative studies, 2 (9%) were mixed methods studies, and 15 (68%) were qualitative studies.

**Table 2:** Characteristics and details of the included studies (in alphabetical and date order).

Study	Aim (relevant to this review)	Intervention details	Design	Data collection	Sample	Analysis
Asiri et al [32]	To explore how social media users in the Arab world share sensitive health information through Facebook	Asynchronous forum, Facebook platform, Arabic only	Qualitative	Retrieval of user-generated messages	Not specified	Content analysis of 271 messages posted between December 1, 2010, and December 1, 2014
Bussone et al [33]	To investigate how people living with HIV and AIDS use a web-based community in terms of health information sharing	Public, asynchronous forum moderated by members	Qualitative	Retrieval of user-generated messages	Not specified	Thematic analysis of 252 messages
Coursaris and Liu [14]	To explore the exchange of social support within an HIV and AIDS–related online support group	Public, asynchronous forum	Qualitative	Retrieval of user-generated messages	Not specified	Content and thematic analysis of 5000 messages
Dong et al [34]	To explore whether social media could be used to study people living with HIV and AIDS and their needs and web-based habits	Asynchronous forum, Baidu Tieba platform, Chinese	Qualitative	Retrieval of user-generated messages	Not specified	Content analysis of 2340 messages posted between 2015 and 2017
Flickinger et al [35]	To examine how social support is exchanged as well as the benefits or limitations of a web-based community message board for people living with HIV	Private, asynchronous forum moderated by professionals	Qualitative	Retrieval of user-generated messages; semistructured interviews	55 HIV-positive individuals (mean age 39, SD 11.68 years; n=37, 67% male)	Content analysis of 840 messages and qualitative analysis of interviews
Flickinger et al [36]	To evaluate content posted to a web-based community message board for people living with HIV	Private, asynchronous forum moderated by professionals	Qualitative	Retrieval of user-generated messages	38 HIV-positive individuals (mean age 34.1, SD 11.5 years; n=28, 74% male)	Content analysis of 840 messages posted during the first 8 months of a community message board
Flickinger et al [37]	To understand the discussion of stigma within the web-based community message board and to evaluate participants’ stigma levels at the 12-month follow-up	Private, asynchronous forum moderated by professionals	Mixed methods	Retrieval of user-generated messages; longitudinal survey	77 HIV-positive individuals (n=49, 64% male)	Content analysis of 394 messages; change in HIV-related stigma at 12 months
Gadgil et al [38]	To explore quality factors that may underpin the success of an online support group for people living with HIV	Private, asynchronous Facebook platform	Qualitative	Web-based semistructured interviews	32 HIV-positive individuals	Grounded theory analysis of interviews
Gaysynsky et al [39]	To examine the types of interaction that occurred within a Facebook online support group	Private, asynchronous forum, Facebook platform	Qualitative	Retrieval of user-generated messages	43 HIV-positive individuals (n=28, 65.1% male)	Content analysis of 3838 messages posted between March 1, 2011, and July 1, 2012
Guo and Goh [40]	To study changes in the composition of socioemotional and informational content in an HIV and AIDS online support group over time	Private, asynchronous, Sina Weibo microblogging platform moderated by the founding member	Qualitative	Retrieval of user-generated messages	Not specified	Content analysis of messages posted during the first 10 weeks (n=1277) of the online support group’s existence and the last 10 weeks (n=966) of the data collection period
Han et al [41]	To explore how people who have self-labeled their HIV identity use social media	Public, asynchronous, Sina Weibo microblogging platform	Qualitative	Retrieval of user-generated messages	Not specified	Deductive thematic analysis of 1507 messages posted between January 1, 2015, and May 22, 2015
Han et al [42]	To examine whether individuals living with HIV and AIDS perceive higher levels of social support via Weibo than from offline relationships and whether perceived online support is associated with enacted social support and predictive of better health outcomes	Public, asynchronous, Sina Weibo microblogging platform	Quantitative	Cross-sectional web-based survey	432 HIV-positive individuals (mean age 29.2, SD 5.87 years; n=414, 95.8% male), years since HIV diagnosis (mean 2.69, range 0.1-11.4 years)	Multivariate analysis
Lai and Peirce [43]	To explore types of social support observed within public HIV and AIDS–related online support groups	Public, asynchronous forums (N=6)	Qualitative	Retrieval of user-generated messages	Not specified	Content analysis of 113 messages posted within the previous 30 days
Maestre et al [44]	To examine social support exchanges within HIV and AIDS–related online support groups	Public, asynchronous forums (N=4) moderated by professionals and members	Qualitative	Retrieval of user-generated messages	233 individuals	Content analysis of 400 messages (ie, the most recent 100 messages from each forum)
Mo and Coulson [45]	To examine the type of social support contained within messages posted to an HIV and AIDS–related online support group	Public, asynchronous forum (N=1)	Qualitative	Retrieval of user-generated messages	171 individuals	Content analysis of 85 threads randomly selected from all threads (n=342; 5230 messages) posted between June 1, 2006, and June 30, 2006
Mo and Coulson [46]	To explore whether differences exist between lurkers and posters in their use of HIV and AIDS–related online support groups, experience of empowering processes, and outcomes and satisfaction	Public, asynchronous forums (N=6), moderated	Quantitative	Cross-sectional web-based survey	340 HIV-positive individuals (mean age 47.8, SD 10.6 years; n=283, 83.7% male), years since HIV diagnosis (mean 11.8 years)	Multivariate analysis
Mo and Coulson [47]	To explore the use of online support groups and association with health status, coping, and social support among individuals living with HIV and AIDS	Asynchronous forum (N=1)	Quantitative	Cross-sectional web-based survey	640 HIV-positive individuals (mean age 45.52, SD 9.26 years; n=525, 82.4% male), years since HIV diagnosis (mean 9.69, SD 6.80 years)	Classification of online support group users into (1) nonusers, (2) infrequent users, and (3) frequent users followed by multivariate analysis
Mo and Coulson [48]	To explore the mechanisms through which participation in HIV and AIDS–related online support groups may promote patient empowerment	Public, asynchronous forums (N=6), moderated	Quantitative	Cross-sectional web-based survey	340 HIV-positive individuals (mean age 47.8, SD 10.6 years; n=283, 83.7% male), years since HIV diagnosis (mean 11.8 years)	Structural equation modeling
Mo and Coulson [49]	To examine the relationship among online support group use, patient empowerment, and psychological outcomes for individuals living with HIV and AIDS	Public, asynchronous forums (N=6), moderated	Quantitative	Cross-sectional web-based survey	340 HIV-positive individuals (mean age 47.8, SD 10.6 years; n=283, 83.7% male), years since HIV diagnosis (mean 11.8 years)	Structural equation modeling
Mo and Coulson [29]	To explore the presence of potentially empowering and disempowering processes and outcomes within HIV and AIDS–related online support groups	Public, asynchronous forums (N=4), moderated	Qualitative	Cross-sectional web-based survey	115 HIV-positive individuals (mean age 45.92, SD 9.96 years; n=102, 88.7% male), years since HIV diagnosis (mean 10.59, SD 13.77 years)	Thematic analysis of open-ended responses coding for both empowering and disempowering processes as well as outcomes
Peterson [50]	To explore how an HIV and AIDS–related online support group delivers positive social support and builds community	Private email listserv, moderated	Qualitative	Retrieval of user-generated messages	Not specified	Grounded theory analysis of 1870 messages posted over a 2-month period
Shi and Chen [51]	To explore the types of social support observed within a Chinese HIV and AIDS–related online support group	Public, asynchronous, Sina Weibo microblogging platform	Qualitative	Retrieval of user-generated messages	Not specified	Content analysis of all 7215 messages posted since its creation on January 18, 2011, as of September 14, 2012

Of the 6 quantitative designs (n=5, 83% quantitative studies and n=1, 17% mixed methods studies), only 1 (17%) was longitudinal and measured changes in HIV-related stigma over time [37]. The other 83% (5/6) of the quantitative studies were cross-sectional, measuring engagement with online support groups at only 1 time point [42,46-49]. The qualitative studies and the qualitative component of the mixed methods studies undertook an analysis of user-generated content [14,32-34,36,37,39-41,43-45,50,51] or interviewed online support group members [35,38], with 6% (1/17) of the studies analyzing the content of responses to open-ended questions [29].

As can be seen in Table 2, publication dates for the included studies ranged from 2007 to 2019; 45% (10/22) of the studies were published within the last 5 years, with 36% (8/22) being published within the last 10 years and 18% (4/22) being >10 years old. As judged by the address for the lead or corresponding author, the research teams were based in the United States (9/22, 41%), China or Hong Kong (5/22, 23%), the United Kingdom (4/22, 18%), Singapore (2/22, 9%), Australia (1/22, 5%), and Saudi Arabia (1/22, 5%). Online support groups included public and private asynchronous forums, microblogging websites, Facebook groups, and an email listserv. Across the 22 included studies, a total of 27,421 user-generated messages were analyzed along with 87 interviews and 1527 survey responses.

### Synthesis of Results

#### Research Question 1: Does Engagement With Online Peer Support Groups Improve Psychosocial Well-being and Illness Management in Those Diagnosed With HIV and AIDS?

##### Overview

To address this research question, data were extracted and synthesized from 100% (5/5) of the quantitative studies [42,46-49] and the quantitative data from 50% (1/2) of the mixed methods studies [37]. These data are presented in Table 3 synthesized into a single theme (*Positive and negative associations between engagement and psychosocial outcomes*). Next, we extracted data from 27% (4/15) of the qualitative studies [14,35,38,39] and the qualitative data from 50% (1/2) of the mixed methods studies [29]. We synthesized this into a single theme (*Connecting with similar others*) capturing the positive impact of engagement on psychosocial well-being and illness management.

**Table 3:** Variables, measures, and key findings from the included quantitative and qualitative studies.

Study type and study			Variables and measures		Summary of key findings
**Longitudinal**					
	Flickinger et al [37]	HIV-related stigma: 40-item Berger Stigma Scale (possible score range from 40 to 160)		Baseline: mean 102.94 (SD 18.26); 12 months: mean 98.73 (SD 15.08). There was a trend toward reduced stigma, with a mean change of −3.9 (95% CI −8.1 to 0.2), but it was not statistically significant (*P*=.06). Among those who posted, posters of content unrelated to stigma had a mean change in stigma scores of −3.3 (SD 12.7) compared with −5.1 (SD 17.2) for posters of stigma-related content. There was a trend toward more improvement in stigma scores with posting vs not posting and with posting about stigma vs other content, though these differences were not statistically significant (*P*=.50 and *P*=.72, respectively; 1-way ANOVA *F* test).	
**Cross-sectional**					
	Han et al [42]	OSG^a^ use: length of use (years) and frequency of Weibo use; health information: date of HIV diagnosis, recent CD4 cell counts, and HIV status disclosure; enacted giving social support: 5 items; enacted receiving social support: 4 items; perceived offline social support: 12-item Multidimensional Scale of Perceived Social Support; perceived online social support: 8-item modified Multidimensional Scale of Perceived Social Support; subjective well-being: 5-item Satisfaction with Life Scale; adherence to ART^b^: 6 items; risky sexual behavior: 5 items		Perceived online social support was associated with employment status (employed people had higher levels of support compared with unemployed people), CD4 cell counts (those with lower CD4 cell counts perceived more support), and perceived offline support (higher offline support was associated with higher online support). People living with HIV and AIDS perceived higher levels of social support from Weibo than from offline support (from family and friends).	
	Mo and Coulson [46]	OSG use: length of use (months) and days and hours per average week; satisfaction with online support group experience: 4 items; empowering processes: 43-item scale, 4 processes (receiving useful information, receiving social support, finding positive meaning, and helping others); self-care self-efficacy: 29-item Strategies Used by People to Promote Health Scale; loneliness: 10-item UCLA^c^ Loneliness Scale; optimism: 10-item Life Orientation Test-Revised; coping: 28-item Brief COPE^d^; depression: 20-item Center for Epidemiological Studies Depression Scale-Revised; quality of life: 35-item Medical Outcome Study HIV Health Survey		Compared with posters, members who only read the messages (“lurkers”) scored lower in receiving social support and receiving useful information in empowering processes and lower in satisfaction with their relationship with group members. They also scored higher in distraction and lower in planning on the Brief COPE. In addition, they scored lower in social function and higher in energy. There were no significant differences in self-care self-efficacy, loneliness, depression, or optimism between posters and “lurkers.”	
	Mo and Coulson [47]	Medical history: time since diagnosis, disease stage, and recent CD4 cell count; OSG use: hours in the previous month; health status: 36-item Medical Outcomes Study Short Form 36; coping: 28-item Brief COPE; perceived social support: 19-item Medical Outcomes Study Social Support Survey		Frequent users reported poorer health than nonusers. In addition, both frequent and infrequent users scored higher in planning, active coping, instrumental support, and emotional support coping on the Brief COPE. No significant difference was found for social support.	
	Mo and Coulson [48]	OSG use: length of use and days and hours per average week; empowering processes: 43-item scale, 4 processes (receiving useful information, receiving social support, finding positive meaning, and helping others); self-care self-efficacy: 29-item Strategies Used by People to Promote Health Scale; coping: 28-item Brief COPE; quality of life: 35-item Medical Outcome Study HIV Health Survey		The use of online support groups was significantly related to higher levels of all 4 empowering processes. Receiving useful information and finding positive meaning were related to higher levels of adaptive coping and lower levels of maladaptive coping, whereas receiving social support and helping others were related to higher levels of self-care self-efficacy, which in turn was related to higher levels of adaptive coping and lower levels of maladaptive coping. Finally, higher levels of adaptive coping and lower levels of maladaptive coping were related to better quality of life.	
	Mo and Coulson [49]	OSG use: length of use and days and hours per average week; empowering processes: 43-item scale, 4 processes (receiving useful information, receiving social support, finding positive meaning, and helping others); loneliness: 10-item UCLA Loneliness Scale; optimism: 10-item Life Orientation Test-Revised; depression: 20-item Center for Epidemiological Studies Depression Scale-Revised		Online support group use was positively related to empowering processes, which in turn was positively related to optimism toward life. Optimism was negatively related to loneliness and depression. Loneliness was also positively related to depression.	
**Qualitative**					
	Coursaris and Liu [14]	N/A^e^		Members of the group shared their personal conditions, thoughts, and feelings related to HIV with others as well as expressing gratitude or sending congratulations. This disclosure and actions served to promote reciprocal disclosure and promote group ties. Consequently, members felt better about themselves.	
	Flickinger et al [35]	N/A		The community message board helped individuals connect with others going through a similar experience and fostered a sense of universality. The mutual exchange of support between members was also described as beneficial, with both informational and emotional support being particularly helpful in terms of outlook.	
	Gadgil et al [38]	N/A		Sharing experiences of stigma and memories of shame, guilt, and pain promoted a sense of camaraderie that mitigated the negative impacts of both felt and enacted stigma.	
	Gaysynsky et al [39]	N/A		Participation in an online support group helped members feel like they were being treated as an equal.	
	Mo and Coulson [29]	N/A		Engagement with online support groups was associated with six empowering processes: (1) exchanging information, (2) sharing experiences, (3) connecting with others, (4) encountering emotional support, (5) finding recognition and understanding, and (6) helping others. Six empowering outcomes arising from engagement with online support groups were identified: (1) increased optimism, (2) emotional well-being, (3) social well-being, (4) being better informed, (5) improved disease management, and (6) feeling confident in the relationship with physicians.	

^a^OSG: online support group.

^b^ART: antiretroviral therapy.

^c^UCLA: University of California, Los Angeles.

^d^COPE: Coping Orientation to Problems Experienced.

^e^N/A: not applicable.

##### Positive and Negative Associations Between Engagement and Psychosocial Outcomes

Of the 6 quantitative studies, only 1 (17%) was a longitudinal investigation. Flickinger et al [37] examined changes in HIV-related stigma over time (ie, at baseline and after 12 months). Although there was a reduction in HIV-related stigma, this was not significant.

A total of 23% (5/22) of the studies adopted a cross-sectional approach when considering the relationship between engagement with online support groups and psychosocial well-being and illness management. Using the same data set, 9% (2/22) of the studies [46,49] found that engagement with online support groups was positively associated with greater exposure to a number of “empowering processes.” These, in turn, were positively associated with adaptive coping, self-care self-efficacy, optimism, and quality of life [46] and lower levels of loneliness and depression [49]. A paper from this data set [46] considered whether these differences would exist when engagement was viewed in terms of “lurking” (ie, not posting any messages) versus “posting,” but no differences were found in terms of self-care self-efficacy, loneliness, depression, or optimism. Similarly, no differences were found between frequent and infrequent users of online support groups in relation to perceived social support [47]. However, other sociodemographic and medical factors were predictive of perceived online support in 5% (1/22) of the studies [42]. Indeed, this study found that people living with HIV and AIDS perceived higher levels of social support from their online support group than from their offline networks.

##### Connecting With Similar Others

This theme captured the way in which engagement with HIV and AIDS–related online support groups was considered beneficial to individuals in terms of improving their psychosocial well-being and illness management. The qualitative studies illuminated how online support groups provided an opportunity to meet and interact with other individuals who were currently experiencing or had in the past experienced similar issues and challenges [14,29,35,38,39]. Through the sharing of personal experiences, thoughts, and feelings as well as messages offering congratulations, gratitude, and acknowledgment, a sense of camaraderie and group cohesion was developed. In addition, 5% (1/22) of the studies described how the exchange of informational and emotional support was helpful to group members [35]. Together, the ability to connect and engage in mutually supportive interaction helped individuals in terms of their self-worth [14,39] and outlook as well as mitigating the negative impacts of stigma [38]. A total of 5% (1/22) of the studies [29] described how this mutually supportive interaction also benefited individuals in terms of better illness management and improved confidence when communicating with health care professionals.

#### Research Question 2: Are There Any Negative Aspects of Online Support Groups Experienced by Individuals Living With HIV and AIDS? If So, What Are These?

##### Overview

To address this research question, data were extracted and synthesized from 33% (5/15) of the qualitative studies [29,32,35,36,39] and the qualitative data from 50% (1/2) of the mixed methods studies [37]. Three themes capturing the negative aspects of online support groups experienced by users were generated from the findings (Table 4): (1) challenging behavior, (2) difficult content, and (3) negative consequences of the web-based platform.

**Table 4:** Qualitative and descriptive findings regarding the negative aspects of engagement.

Study	Negative aspects of engagement: themes
Asiri et al [32]	Negative judgment or attribution of blame Concerns about privacy and disclosure
Flickinger et al [35]	Challenging or negative content (complaining, suicidal ideation, attacking, vulgarity, poor language, taboo topics, excessive personal information, and religiosity) Feelings of obligation or keeping up to date with conversations Lack of activity or immediacy of feedback Lack of anonymity Being unable to connect physically Feeling like an outsider in the community Potential of lost relationships once the research study was completed
Flickinger et al [36]	The impact of negative posts—disturbing or disruptive to the community (eg, posts expressing suicidal thoughts or mental health concerns)
Flickinger et al [37]	Negative thread interactions—contained posts expressing strong emotions on the CMBa or posts containing a negative reaction to another member’s post
Gaysynsky et al [39]	Interpersonal conflict (disrespectful, sarcastic, unkind, or argumentative); statements that expressed being hurt, distressed, or angered by other members of the group and statements that demonstrated disagreement, tension, or antagonism
Mo and Coulson [29]	Being unable to connect physically Inappropriate behavior on the web (inappropriate, disrespectful, attacking, or ridiculing) Declining real-life relationships (overreliance on web-based relationships) Informational overload and misinformation

^a^CMB: community message board.

##### Challenging Behavior

This theme was concerned with behavior of group members that was considered inappropriate. Some studies (2/6, 33%) described how members felt that the behavior of others was inappropriate, unkind, disrespectful, or attacking or was trying to ridicule others [29,39].

##### Negative Content

Several studies (4/6, 67%) reported how the content posted to the online support groups was negative or difficult to deal with. For example, Flickinger et al [35] reported instances of complaining, vulgarity, bad language, taboo topics, excessive personal information, and religiosity. Furthermore, the challenge of being exposed to posts that were disturbing or disruptive to the community, such as posts expressing suicidal thoughts or mental health concerns, was highlighted [36]. Asiri et al [32] also reported instances in which some of the content posted on the web appeared to be judging or ascribing blame to individuals for their HIV status.

##### Negative Consequences of the Web-Based Platform

A range of issues was identified that reflected the negative consequences arising from the platform underpinning the online support group. For example, 33% (2/6) of the studies noted the difficulties arising from the fact that group members were not physically copresent [35], and this presented challenges in terms of forming web-based relationships [29]. However, some concerns were also expressed regarding overreliance on these web-based relationships and the potential for a decline in real-world relationships as a possible consequence [29]. Concerns were also expressed regarding the quantity and quality of information exchanged between group members. For example, Flickinger et al [35] noted the difficulties experienced by individuals when trying to keep up with conversations on the web, whereas other studies (1/6, 17%) reported difficulties regarding both information overload and accuracy [29]. Flickinger et al [35] described problems concerning a lack of activity or immediacy of feedback on members’ posts. In addition, they noted concerns regarding the lack of anonymity in group participation. Asiri et al [32] also identified concerns regarding privacy and disclosure in their study.

#### Research Question 3: What Types of Social Support Are Exchanged Within Online Support Groups for Individuals Affected by HIV or AIDS?

To address this research question, data were extracted and synthesized from 73% (11/15) of the qualitative studies [14,33-35,39-41,43-45,51], which analyzed user-generated content for evidence of social support exchange. These are presented in Table 5. Most studies (8/11, 73%) included sufficient detail to populate Table 5 but, in some instances, we undertook a simple calculation from the available data to report support requests or provision.

**Table 5:** Social support exchange.

Study	Overall	Social support requests (% of total requests)	Social support provision (% of total provision)
Bussone et al [33]	252/2455 (10.26%) messages either asked or responded to questions about personal health information; 60/2455 (2.4%) messages requested social support; 192/2455 (7.8%) messages provided social support	60/2455 (2.4%) messages that contained 77 different questions	Informational support: 176/192 (91.7%) Emotional support: 56/192 (29.2%) Esteem support: 15/192 (7.8%) Network support: 15/192 (7.8%) Tangible support: 1/192 (0.5%)
Coursaris and Liu [14]	815/5000 (16.3%) messages requested social support; 2310/5000 (41.6%) messages provided social support 87% of messages included 1 type of support, 12% included 2 types, and 1% included 3 types	Informational support: 626/815 (76.8%) Emotional support: 154/815 (18.9%) Network support: 78/815 (9.6%) Esteem support: 26/815 (3.2%) Tangible assistance: 13/815 (1.6%)	Informational support: 1458/2310 (63.1%) Emotional support: 646/2310 (28%) Esteem support: 294/2310 (12.7%) Network support: 260/2310 (11.3%) Tangible assistance: 27/2310 (1.2%)
Dong et al [34]	726/2340 messages (31.03%) included social support; 559/2340 (23.9%) messages requested social support; 167/2340 (7.1%) messages provided social support	Request for friendship: 436/559 (78%)	Sharing knowledge: 43/167 (25.8%)
Flickinger et al [35]	115/840 (14%) messages requested social support; 433/840 (52%) messages provided social support	Emotional support: 85/840 (10.1%) Informational support: 30/840 (3.6%) No evidence of instrumental support	Emotional support: 178/433 (41.1%) Network support: 115/433 (26.6%) Esteem support: 77/433 (17.8%) Informational support: 55/433 (12.7%) Instrumental support: 8/433 (1.8%)
Gaysynsky et al [39]	255/3838 (6.6%) messages requested social support; 578/3838 (15.1%) messages provided social support	Emotional support: 82/255 (32.2%) Network support: 80/255 (31.4%) Informational support: 56/255 (22%) Tangible assistance: 37/255 (14.5%) Esteem support: 21/255 (8.2%)	Esteem support: 259/578 (44.8%) Emotional support: 149/578 (25.8%) Network support: 113/578 (19.6%) Informational support: 83/578 (14.4%)
Guo and Goh [40]	1277 messages posted during the first 10 weeks; 966 messages posted during the last 10 weeks Socioemotional messages—481 (37.67%) to 494 (51.14%)—exceeded informational messages—796 (62.33%) to 472 (48.86%)—over time	—^a^	—
Han et al [41]	135/1507 (9%) messages requested social support; 603/1507 (40%) messages provided social support	Emotional support: 57/135 (42.2%) Informational support: 52/135 (38.5%) Others: 26/135 (19.3%)	HIV and AIDS–related: 104/603 (17.2%) Daily life events: 499/603 (82.7%)
Lai and Peirce [43]	11/113 (9.7%) messages requested social support; 104/113 (92%) messages provided social support	Emotional support: 5/11 (45.5%) Informational support: 3/11 (2.7%) Instrumental support: 3/11 (2.7%)	Informational support: 61/104 (58.7%) Emotional support: 34/104 (32.7%) Instrumental support: 9/104 (8.7%)
Maestre et al [44]	400 messages yielding 525 utterances; 292/525 (55.6%) utterances requested social support; 233/525 (44.4%) utterances provided social support	Informational support: 165/292 (56.5%) Emotional support: 108/292 (37%) Esteem support: 13/292 (4.5%) Network support: 6/292 (2.1%) No evidence of tangible support	Informational support: 126/233 (54.1%) Emotional support: 83/233 (35.6%) Esteem support: 17/233 (7.3%) Network support: 7/233 (3%) No evidence of tangible support
Mo and Coulson [45]	986/1138 (86.6%) messages contained at least one type of social support	—	Informational support: 466/986 (47.3%) Emotional support: 369/986 (37.4%) Esteem support: 130/986 (13.2%) Network support: 72/986 (7.3%) Tangible assistance: 10/986 (1%)
Shi and Chen [51]	5589/7215 (77.46%) messages included social support; 1588/7215 (22.01%) messages requested social support; 4001/7215 (55.45%) messages provided social support	Informational support: 1051/1588 (66.2%) Instrumental support: 319/1588 (20.1%) Emotional support: 218/1588 (13.7%)	Informational support: 2528/4001 (63.2%) Emotional support: 1405/4001 (35.1%) Instrumental support: 68/4001 (1.7%)

^a^Not available.

All studies used the message as the level of analysis except for the study by Maestre et al [44], which used the utterance. All studies (11/11, 100%) used a deductive analytical approach, with 55% (6/11) of the studies [14,33,35,39,44,45] using the Social Support Behavior Code developed by Cutrona and Suhr [52] as their underpinning coding framework. From the remaining studies, 9% (1/11) [41] combined the Social Support Behavior Code with interaction process analysis, 9% (1/11) [43] used a social support conceptual framework developed by House and Kahn [53], and 9% (1/11) [51] were guided by the typology described by Wright et al [54]. A further 18% (2/11) of the studies [34,41] stated that researchers with experience in public health developed the categories of social support to be coded.

A total of 82% (9/11) of the studies distinguished between social support requests and provision [14,33-35,39,41,43,44,51]. Of these 9 studies, 2 (22%) [34,44] reported a higher percentage of support requests than support provision, whereas 7 (78%) [14,33,35,39,41,43,51] reported a higher percentage of messages classified as providing social support. In terms of support requests, 36% (4/11) of the studies reported emotional support as being the most frequently requested [35,39,41,43], and 27% (3/11) reported informational support as being the most frequently requested [14,44,51]. For support provision, 55% (6/11) of the studies [14,33,43-45,51] reported informational support as being the most prevalent type of social support offered, with 9% (1/11) [35] reporting emotional support as being the most common and 9% (1/11) [39] reporting esteem support as being the most common. Only Guo and Goh [40] considered how the exchange of social support changed over time. They found that socioemotional messages exceeded informational support messages over time.

## Discussion

### Principal Findings

Online support groups offer new opportunities for those living with HIV and AIDS to access information, advice, and mutual peer support. To our knowledge, this is the first scoping review to synthesize the evidence regarding HIV or AIDS–related online support groups. Our review had three aims; we sought to determine (1) whether engagement with online peer support groups improved psychosocial well-being and illness management for those living with HIV and AIDS; (2) whether there existed any negative aspects of online support groups experienced by individuals living with or affected by HIV and AIDS and, if so, what were they; and (3) what types of social support were exchanged within online support groups for individuals living with or affected by HIV and AIDS. We addressed the first research question by synthesizing the findings of a range of published quantitative, mixed methods, and qualitative studies. The second and third research questions were answered by synthesizing the findings of published qualitative studies.

In terms of our first research question, no randomized controlled trials of the impact of engagement with HIV and AIDS–related online support groups on psychosocial well-being and illness management were identified. However, we did extract relevant findings from quantitative and qualitative studies as well as the quantitative component of a mixed methods study. There was limited quantitative evidence that engagement with online peer support groups improves psychosocial well-being. Indeed, the only longitudinal study conducted over a 12-month period [37] reported no changes in HIV-related stigma. All the cross-sectional studies (5/22, 23%) reported an association between engagement with HIV or AIDS–related online support groups and psychosocial well-being and illness management [42,46-49]; however, it is difficult to make any causal inference owing to the cross-sectional nature of the studies. In terms of qualitative evidence, benefits arising from engagement with HIV and AIDS–related online support groups were reported across all the studies (15/22, 68%), in particular the psychosocial benefits of individuals coming together on the web and sharing their experiences together with the mutual exchange of support. Mo and Coulson [29] described a range of “active ingredients” that may benefit individuals who engage with HIV or AIDS–related online support groups. These included exchanging information, sharing experiences, connecting with others, encountering emotional support, finding recognition, and understanding and helping others. In turn, they identified several psychosocial outcomes that may arise from engagement, including increased optimism and control over the future, improved emotional and social well-being, being better informed, improved coping, and feeling more confident in their relationship with health care professionals.

In relation to our second research question, there were 27% (6/22) of studies reporting qualitative data on the negative aspects of engagement with online support groups. We identified 3 key problematic issues. The first revolved around the challenging behavior of other group members, particularly in terms of interpersonal conflict. Next, we identified the challenge of negative content and how group members could be exposed to online material that was difficult to read. Finally, we noted negative experiences that may arise from the unique features (eg, text-based and asynchronous) of the web-based platforms used to support the online support groups. Specifically, we noted difficulties in forming online relationships but also instances of overreliance on these relationships, which may then negatively affect offline relationships. We also identified concerns regarding both information quantity and quality. These various concerns have been reported elsewhere in the literature [24,27,55] and are potentially serious in nature. However, we currently have little understanding of the long-term impact of these negative experiences on group members. Moving forward, these findings do suggest that online support group moderators or administrators may play a crucial role in achieving the aims of the support group and safeguarding its membership.

Half of the studies included in our review (11/22, 50%) addressed the third research question, which considered the types of social support exchanged within online support groups for individuals living with or affected by HIV and AIDS. In total, 100% (11/11) of the qualitative studies used a deductive analytic approach with a social support framework (or typology) to guide the analysis, with most studies (6/11, 55%) using the Social Support Behavior Code developed by Cutrona and Suhr [52]. There were more studies reporting a higher proportion of support provision than of requests. This may be explained by the asynchronous text-based platforms that were used in the studies. On these platforms, conversational threads are developed by individual group members posting a message and other group members posting replies. Our findings also revealed that emotional and informational support were the types most frequently requested but, in terms of provision, informational support was the most common type of support reported. These findings are consistent with the results of a meta-analytic review of 41 published studies that reported informational and emotional support messages as the most prevalent within health-related online support groups [56].

### Strengths and Limitations of This Review

It is important to highlight the strengths of this scoping review. Most notably, we identified, described, and synthesized data from quantitative, qualitative, and mixed methods studies and considered the guidance set out by Booth et al [57] to support this process. In doing so, we believe this has provided the reader with a richer and more holistic insight into the role of online support groups for individuals affected by HIV and AIDS. However, there are also some limitations that should be considered. First, although we searched 7 databases, it is possible that we failed to identify some relevant studies. However, to mitigate this, we also searched Google Scholar as well as hand searching the reference lists of all the included studies. This yielded additional studies that were included in our review. Second, our review may have introduced bias through the inclusion of only studies published in English; thus, we may have overlooked studies published in other languages. Finally, our review identified a few quantitative studies, and those that were included were mostly cross-sectional surveys. Therefore, it becomes difficult to draw definitive conclusions, particularly in relation to the psychosocial benefits arising from engagement with HIV and AIDS–related online support groups.

### Recommendations for Future Research

This scoping review has revealed that there exists a growing and diverse body of literature that considers the role of online support groups for people affected by HIV and AIDS. This literature includes quantitative, qualitative, and mixed methods research designs. However, our review also pinpointed specific areas for future research to advance our understanding of the role and impact of online support groups for individuals affected by HIV and AIDS. First, to assess the impact of engagement more fully, future research efforts should seek to develop more robust research designs, including randomized controlled trials and longitudinal studies on both the benefits and harms of engagement. This work should also seek to consider a broader array of psychosocial as well as illness-related outcome measures. Second, as these online support groups may be supported by a range of platforms, future research should seek to explore how the affordances of each platform may influence both engagement and outcomes. Third, our review noted that online support group moderators may play a pivotal role in promoting the aims of the group as well as safeguarding its membership. Therefore, further research examining the function and effectiveness of moderator web-based behavior is warranted. Fourth, as the exchange of informational support appears widespread within online support groups for HIV and AIDS, future work may seek to determine the accuracy of any medical-related information shared and the extent to which it may affect the coping strategies and behaviors of members.

### Conclusions

Online support groups provide an opportunity for individuals affected by HIV and AIDS to engage in mutual support. This engagement may be associated with improved illness management as well as a range of beneficial psychosocial outcomes. However, members may experience negative aspects of the online support groups, particularly in terms of interpersonal conflict with other members and content that is challenging. Online support groups for HIV and AIDS can provide a valuable opportunity to both seek and provide social support, notably informational and emotional support.

## Appendix

Multimedia Appendix 1 Example of MEDLINE search strategy from April 2022.

Multimedia Appendix 2 PRISMA (Preferred Reporting Items for Systematic Reviews and Meta-Analyses) checklist.

## Abbreviations

PRISMA: Preferred Reporting Items for Systematic Reviews and Meta-Analyses
