# Supplementary material for: The Role of Online Support Groups in Helping Individuals Affected by HIV and AIDS: Scoping Review of the Literature
Source: J Med Internet Res. 2022 Jul 26;24(7):e27648. doi: 10.2196/27648 (PMC9364165; doi:10.2196/27648)
Supplement: Multimedia Appendix 1 [file jmir_v24i7e27648_app1.doc]

Database(s): **Ovid MEDLINE(R) ALL**1946 to April 27, 2022
Search Strategy:

| **#** | **Searches** | **Results** |
| --- | --- | --- |
| 1 | exp HIV/ | 84970 |
| 2 | exp HIV Infections/ | 232531 |
| 3 | (HIV or Human Immunodeficiency Virus or AIDS or acquired immunodeficiency syndrome).ab,hw,kf,ot,sy,ti,fx,nm,ox,px,rx,ui. | 356631 |
| 4 | 1 or 2 or 3 | 356631 |
| 5 | (online adj4 support group$).ab,hw,kf,ot,sy,ti,fx,nm,ox,px,rx,ui. | 489 |
| 6 | (online support communit* or online communit*).ab,hw,kf,ot,sy,ti,fx,nm,ox,px,rx,ui. | 912 |
| 7 | (discussion forum$ or discussion fora).ab,hw,kf,ot,sy,ti,fx,nm,ox,px,rx,ui. | 740 |
| 8 | bulletin board$.ab,hw,kf,ot,sy,ti,fx,nm,ox,px,rx,ui. | 249 |
| 9 | (chat room$ or chatroom$).ab,hw,kf,ot,sy,ti,fx,nm,ox,px,rx,ui. | 330 |
| 10 | (computer mediated adj3 support).ab,hw,kf,ot,sy,ti,fx,nm,ox,px,rx,ui. | 41 |
| 11 | (internet adj3 support group$).ab,hw,kf,ot,sy,ti,fx,nm,ox,px,rx,ui. | 161 |
| 12 | message board$.ab,hw,kf,ot,sy,ti,fx,nm,ox,px,rx,ui. | 172 |
| 13 | (online adj2 self help).ab,hw,kf,ot,sy,ti,fx,nm,ox,px,rx,ui. | 160 |
| 14 | (web-based adj2 group$).ab,hw,kf,ot,sy,ti,fx,nm,ox,px,rx,ui. | 177 |
| 15 | web group$.ab,hw,kf,ot,sy,ti,fx,nm,ox,px,rx,ui. | 67 |
| 16 | (web-based adj2 communit*).ab,hw,kf,ot,sy,ti,fx,nm,ox,px,rx,ui. | 115 |
| 17 | exp Social Networking/ | 5381 |
| 18 | "social network*".ab,hw,kf,ot,sy,ti,fx,nm,ox,px,rx,ui. | 18573 |
| 19 | 5 or 6 or 7 or 8 or 9 or 10 or 11 or 12 or 13 or 14 or 15 or 16 or 17 or 18 | 21913 |
| 20 | 4 and 19 | 1644 |
